# Structural Design and Synthesis of Novel Cyclic Peptide Inhibitors Targeting *Mycobacterium tuberculosis* Transcription

**DOI:** 10.3390/life12091333

**Published:** 2022-08-28

**Authors:** Filia Stephanie, Mutiara Saragih, Usman Sumo Friend Tambunan, Teruna J. Siahaan

**Affiliations:** 1 Department of Chemistry, Faculty of Mathematics and Natural Sciences, Universitas Indonesia, Jawa Barat 16424, Indonesia; 2 Department of Pharmaceutical Chemistry, School of Pharmacy, The University of Kansas, Lawrence, KS 66045, USA

**Keywords:** tuberculosis, cyclic peptides, molecular simulation, solid-phase peptide synthesis

## Abstract

Tuberculosis (TB) remains one of the deadliest infectious diseases in the world. Although several established antitubercular drugs have been found, various factors obstruct efforts to combat this disease due to the existence of drug-resistance (DR) TB strains, the need for lengthy treatment, and the occurrence of side effects from drug–drug interactions. Rifampicin (RIF) is the first line of antitubercular drugs and targets RNA polymerase (RNAP) of Mycobacterium tuberculosis (MTB). Here, RIF blocks the synthesis of long RNA during transcription initiation. The efficacy of RIF is low in DR-TB strains, and the use of RIF leads to various side effects. In this study, novel cyclic peptides were computationally designed as inhibitors of MTB transcription initiation. The designed cyclic peptides were subjected to a virtual screening to generate compounds that can bind to the RIF binding site in MTB RNAP subunit β (RpoB) for obtaining a new potential TB drug with a safe clinical profile. The molecular simulations showed that the cyclic peptides were capable of binding with RpoB mutants, suggesting that they can be possibility utilized for treating DR-TB. Structural modifications were carried out by acetylation and amidation of the N- and C-terminus, respectively, to improve their plasma stability and bioavailability. The modified linear and cyclic peptides were successfully synthesized with a solid-phase peptide synthesis method using Fmoc chemistry, and they were characterized by analytical HPLC, LC-ESI-MS^+^, and 1H NMR.

## 1. Introduction

Despite being known as an ancient disease, tuberculosis (TB) is still one of the leading causes of mortality worldwide. Approximately 10 million people acquire this disease annually, with more than half of TB patients localized in countries with a high TB disease occurrence [1]. The COVID-19 pandemic also plays a direct impact in backtracking the global effort to combat this disease; this is reflected in increasing deaths from TB in 2020 [2]. Underreported cases will increase the transmittance risk from this pathogen, and at the same time, they can exacerbate the problems facing many countries, compounded with patient’s treatment compliance, drug accessibility, and the complication of the drug regimen [3,4,5]. Drug discovery efforts have been developed to target the *Mycobacterium tuberculosis* (MTB) cell wall, as well as protein and nucleic acid synthesis; the most well-known first line drugs of the past decades are isoniazid (INH) and rifampicin (RIF) [6,7,8].

Here, MTB RNA polymerase (RNAP) facilitates RNA synthesis and is one of the key enzymes for the survival of this pathogen [9,10]. The RNAP protein complex contains several subunits (two copies of α, β, β′, and ω) forming the core enzyme (2αββ′ω), and addition of the sigma factor regulatory protein (σ subunit) helps this enzyme to recognize the DNA promoter and form the holoenzyme (2αββ′ωσ) [11,12,13]. The β and β′ subunits form the claw-like catalytic site for this protein, and they are conserved in various bacterial cells [8]. The MTB RNAP is the target of RIF, with its binding site on the β subunit approximately 12 Å away from the enzymatic active site [14,15,16,17,18]. The RIF works by inhibiting the synthesis of a short nascent RNA during the initiation step through steric clash, and it is highly potent and about 1000-fold more sensitive for MTB RNAP compared to *Escherichia coli* RNAP [19].

Perpetual uses of the first-line antibiotics have triggered bacterial drug-resistance as a pathogen natural response [20,21]. In the case of RIF, bacterial resistance occurs due to mutation on the 81-bp rifampicin resistance determining region (RRDR) of *rpoB* gene, which is coding for the RNAP β subunit (RpoB). Structural alteration of the binding site resulted in declining affinity towards RIF, especially on codon 445 and 450 for MTB RNAP numbering or codon 526 and 531 for *E. coli* RNAP numbering. These mutations are accounted for in approximately 65–86% of the multi-drug resistance from the TB (MDR-TB) clinical isolates, in which S450L is the most common found mutant [6,22,23,24]. While targeting the transcription process remains as an attractive strategy, finding a new potent drug to address this problem is imperative. The new drugs should be able to have a similar or better affinity compared to RIF; however, they should be able to overcome MDR, with lower side effects for patients.

Peptides potentially have several advantages over currently available antimicrobial drug candidates, including high target specificity, low toxicity, and high accessibility compared to other drug classes [25,26,27]. The development of peptide-based drugs however can often be challenging; this is due to undesired properties, such as low stability in formulation and systemic circulation, as well as low oral bioavailability [28]. Structural modifications to alter physicochemical properties to increase membrane permeation can be achieved by forming cyclic peptidomimetics. Peptidomimetic formation can improve oral absorption and avoid enzymatic degradations in the gastrointestinal (GI) tract and systemic circulation [29,30]. In this study, we conducted a computational screening to design novel cyclic peptide inhibitors that target the RIF binding site of MTB RNAP. Five cyclic peptides were found to bind preferably to RIF binding site. These cyclic peptides were synthesized using a solid-phase peptide synthesizer. The crude peptides were purified using semi-preparative reversed-phase HPLC with a C18 column followed by purity analysis using analytical HPLC with a C18 column. The identity of each peptide was characterized by ESI-MS^+^ and ^1^H NMR.

## 2. Materials and Methods

### 2.1. Computational Screening

#### 2.1.1. Preparation of 3D Receptor Structure

The preparation steps were carried out according to our previous study [31]. The 3D structure of the MTB WT transcription initiation complex containing RNA and RIF was obtained from the Protein Data Bank (https://www.rcsb.org/, accessed on 20 August 2021) (PDBID: 5UHC). The MTB RpoB mutant structures were constructed by introducing the mutation with the ‘Protein Builder’ features in a molecular operating environment (MOE) 2014.09. For the protein structure preparation, AMBER10:EHT for protein and nucleic acid was employed with gas phase solvation. Hydrogens and partial charges were fixed before the preparation took place. The ‘LigX’ feature was used for structural refinement with the default parameter. The prepared structures were then saved in a .moe format.

#### 2.1.2. Cyclic Peptide Ligand Database Construction

Analysis of the RIF binding site of RpoB was achieved by characterizing the pocket binding surface. The ‘Surface and Maps’ features of MOE were used to create the visualization of the binding site according to its lipophilicity. The cyclic hexapeptide sequences were determined from the binding pocket characteristics and resulted in 7500 sequences from amino acid combination. The ChemBioDraw Ultra 14.0 (PerkinElmer, Waltham, MA, USA) software was used to generate all cyclic peptide structures with the ‘Biopolymer’ feature, and the 2D structures were then saved in .cdx and .mol formats. To construct the database, all structures were then imported to a .mdb database file and the 3D structures was generated through several preparation steps according to our previous study [32]. Ligands were subjected to a ‘Wash’ protocol to retrieve the protonated states of the compounds, followed by fixing the partial charges and adjustment of the hydrogens and lone pairs for the minimization steps.

#### 2.1.3. Molecular Docking Simulation

Molecular docking simulation between the prepared protein and cyclic peptide library was performed using MOE software. The docking was carried out with gas phase solvation with a placement triangle matcher, by rescoring London dG, and with refinement as the used force field. The screenings were carried out based on the docking scores and root-mean-square deviation values obtained from the rigid and flexible docking steps. The 2D interaction map and 3D visualization were generated using MOE software.

#### 2.1.4. Computational Pharmacological Properties Prediction

Pharmacological properties of the selected cyclic peptide inhibitors were predicted using several computational tools, as follows: Osiris DataWarrior v5.0.0 (Actelion Ltd., Allschwil, Switzerland) [33], ToxTree v3.1.0 (OpenTox, Uppsala, Sweden) [34,35], SwissADME (http://www.swissadme.ch/, Swiss Institute of Bioinformatics, Lausanne, Switzerland. Accessed on 1 October 2021) [36], admetSAR 2.0 (http://lmmd.ecust.edu.cn/admetsar2/, East China University of Science and Technology, Shanghai, China. Accessed on 1 October 2021) [37], and pkCSM (http://biosig.unimelb.edu.au/pkcsm/, University of Melbourne, Melbourne, Australia. Accessed on 1 October 2021) [38].

### 2.2. Solid-Phase Peptide Synthesis

#### 2.2.1. Reagents

A high-loading Rink amide methylbenzhydrylamine (MBHA) resin, Fmoc-L-Cys(Trt)-OH, Fmoc-L-Trp(Boc)-OH, Fmoc-L-Gln(Trt)-OH, Fmoc-L-Tyr(tBu)-OH, Fmoc-L-Leu-OH, Fmoc-L-His(Trt)-OH, Fmoc-L-Phe-OH, Fmoc-L-Lys(Boc)-OH, Fmoc-L-Val-OH, O-(1H-6-Chlorobenzotriazole-1-yl) (HCTU), and N-methyl morpholine (NMM) were purchased from Gyros Protein Technologies, Tucson, AZ, USA. Additionally, N, N-dimethylformamide (DMF), dichloromethane (DCM), acetonitrile, diethyl ether, acetic anhydride, saturated phenol, and ammonium bicarbonate were purchased from Fisher Scientific (Waltham, MA, USA). Piperidine, 2,6-lutidine, trifluoroacetic acid (TFA), and triisopropylsilane (TIPS) were purchased from Sigma-Aldrich (St. Louis, MI, USA).

#### 2.2.2. Peptide Purification and Analysis with HPLC

A Rainin Dynamax HPLC system with a semi-preparative C18 column (Waters xBridge C-18 column; 250 × 19 mm; 5 µm particle size, Waters Corporation, Milford, MA, USA) was used to purify each peptide from the crude product. An Agilent 1100 HPLC system with an analytical microsorb-MV-100-5 C-18 column (250 × 4.6 mm, 5 µm particle size, Agilent Technologies, St. Clara, CA, USA) was used to characterize peptide purity.

#### 2.2.3. Solid Phase Peptide Synthesis

Cyclic hexapeptides were synthesized with Fmoc-amino acids using a Tribute solid-phase peptide synthesizer according to our previous study [39]. The Fmoc-Rink amide MBHA resin was swollen in DMF for 25 min, and Fmoc deprotection was achieved by adding piperidine 20% (*v*/*v*) in DMF to the resin and stirring for 20 min, followed by the washing step with DMF three times. To couple the Fmoc-amino acid, 0.4 M N-methylmorpholine in DMF and HCTU were added to the resin together with the amino acid solution. The mixture was then stirred with vortex and nitrogen bubbling for 40 min, followed by the washing step with DMF three times. Deprotection and coupling cycles were repeated until the last amino acid of the sequence was added. After the final deprotection, acetylation of the peptide N-terminus was carried out with addition of acetic anhydride in DMF followed by vortex mixing under nitrogen for 30 min. After acetylation, the resin was washed with DMF (3 × 5 mL) and DCM (3 × 5 mL). The resin was dried under vacuum for 1 h.

The peptide was cleaved from the resin by adding TFA cocktail (90% TFA, 2.5% water, 2.5% triisopropylsilane, and 5% saturated phenol) for 2 h. Then, the TFA solution was added into cold diethyl ether to precipitate the crude peptide. The solid crude peptide was isolated by centrifugation for 4 min at 3500 rpm and the dried peptide powder was purified using semi-preparative HPLC with a C-18 column. Purified fractions of the linear peptide were combined and concentrated for the cyclization reaction. Cyclization of the linear peptide was carried out by bubbling air into a dilute solution of the peptide in 1 L ammonium bicarbonate buffer at pH = 8.5; this reaction formed an intramolecular disulfide bond between the N-terminus and C-terminus cysteines. The solvent was removed under vacuum followed by repurification of the cyclic peptide using a semi-preparative HPLC system with a C-18 column. The obtained cyclic peptide was characterized with ESI-MS^+^ mass spectrometry, and ^1^H NMR spectra were acquired on a 500 MHz Bruker AVIII spectrometer.

## 3. Results

### 3.1. Computational Screening

#### 3.1.1. Preparation of 3D Receptor Structure

**Figure 1:**
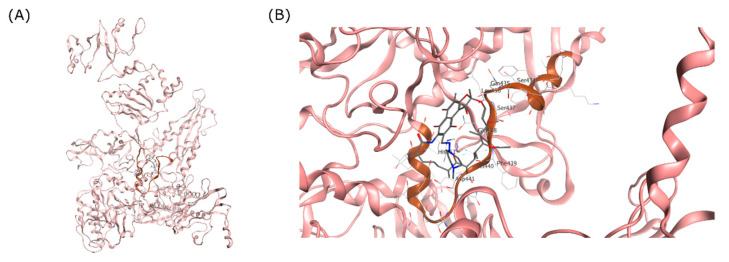
RRDR region of MTB RpoB. (**A**) Visualization of MTB RpoB with RIF binding site shown in a circle. (**B**) The RDRR shown in brown ribbon relative to the RIF molecule on the RIF binding site.

The RpoB structure of the MTB RNAP transcription initiation complex was used as the receptor for the docking simulation. The RIF binding site and RRDR on RpoB are visualized on Figure 1. For this study, the 3D structure of this complex was obtained from the Protein Data Bank for the RpoB X-ray crystal structure with 3.80 Å resolution (PDB ID: 5UHC). Chain C containing the RpoB was preserved from the complex, and all other unwanted chain and small molecules were deleted for computing efficiency purposes. Here, AMBER10 with the extended Hückel theory (EHT) parameterized for proteins, nucleic acids, and small molecules was used as the forcefield. Adjustment of partial charge and 3D structure refinement were achieved using ‘LigX’, including adding hydrogen atoms where protons were missing on the structure. This step is necessary to ensure that the structure is correctly modelled and can accommodate pH-dependent ligand–protein binding during simulation [40].

**Figure 2:**
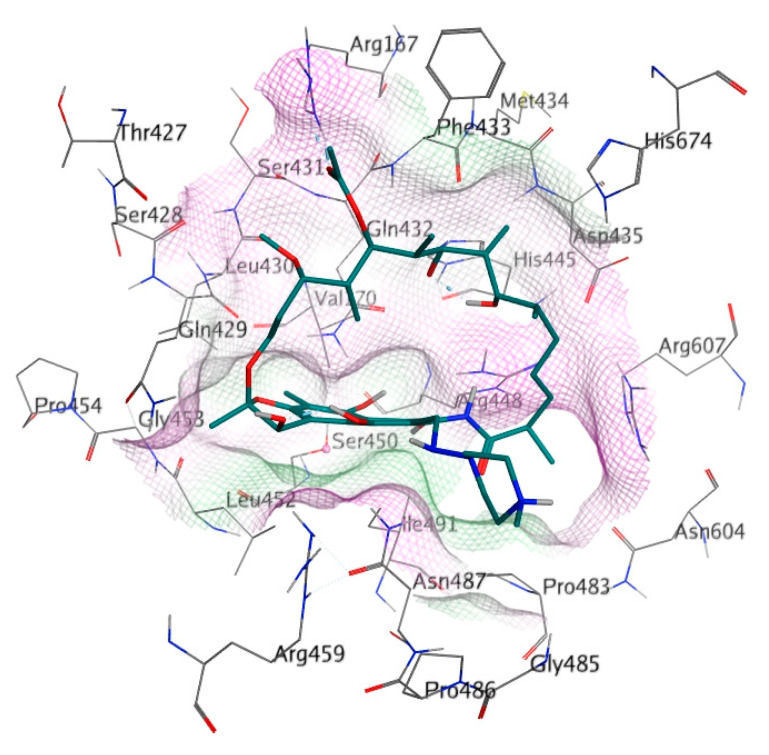
Detailed amino acid labels at RIF binding site on RpoB. The purple and green surfaces indicate hydrophilic and hydrophobic areas, respectively.

The binding site of RIF in the MTB RpoB was used as the docking site for cyclic peptides in this study (Figure 2). The docking site was composed of 26 amino acids, including Arg167, Val170, Thr427, **Ser428**, **Gln429**, **Leu430**, **Ser431**, **Gln432**, **Phe433**, **Met434**, **Asp435**, **His445**, **Arg448**, **Ser450**, Leu452, Gly453, Pro454, Arg459, Pro483, Gly485, Asn487, Pro486, Ile491, Asn604, Arg607, and His674 (Bold letters signify RRDR amino acids). Amino acid mutation was constructed using the ‘Protein Builder’ feature of MOE, followed by structure refinement according to the protocol used for WT RpoB.

#### 3.1.2. Molecular Docking Simulation

A total of 7500 cyclic hexapeptides and RIF were subjected to molecular docking simulation against the prepared receptor structure. Docking was carried out at the RIF binding site of the S450L RpoB and WT RpoB. Rigid docking was employed based on the lock-and-key theorem of protein binding, followed by flexible docking that allows restricted conformational change within the protein and ligand. Retain 100:1 was used on the simulation with ‘triangle matcher’ placement. The screening was conducted based on the RMSD value and the docking scores were generated by the algorithm. The peptides that did not exhibit any possible interaction with S450L RpoB were eliminated through each simulation stage. Rigid docking resulted in 975 potential ligands and, after subjecting them to flexible docking, five ligands with the lowest docking scores were obtained. The interactions between best peptide ligands or rifampicin to RpoB binding site were summarized as docking scores and RMSD in Table 1. From the docking results to S450L RpoB, RIF produced an unacceptable RMSD value, suggesting unfavorable binding to the active site of the mutant S450L RpoB. This is one of the possible reasons that the RpoB mutant strain is resistant to RIF. In contrast, docking of RIF to RpoB WT gave a low RMSD value and the lowest docking score, in agreement with RIF efficacy towards RpoB WT.

**Figure 3:**
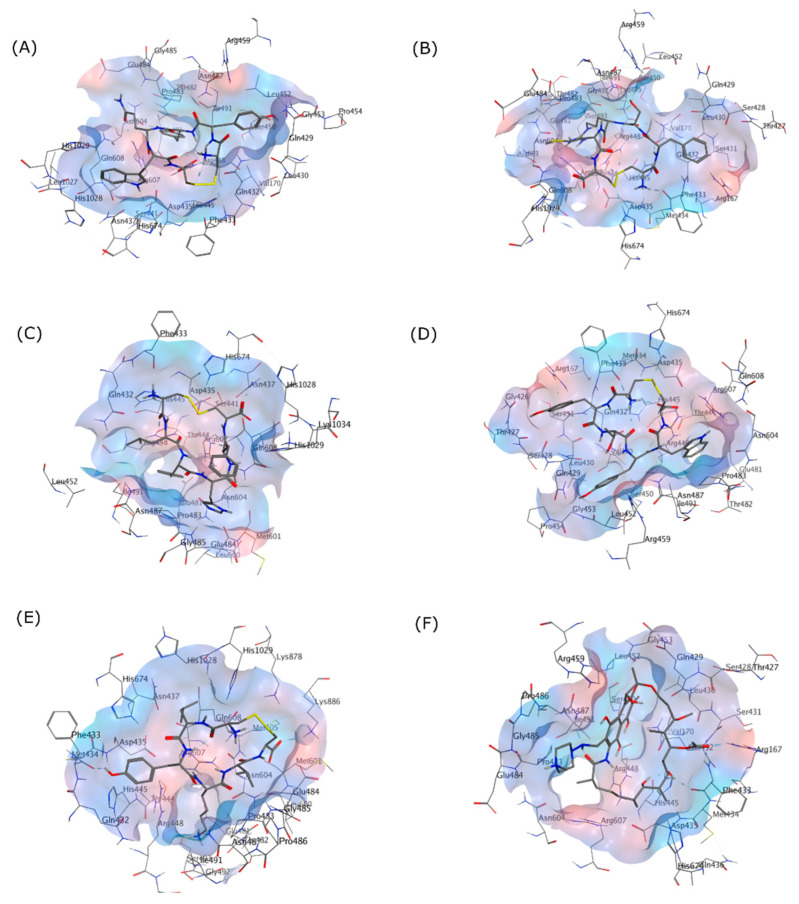
3D Interactions of (**A**) (Cyclo-1,6)CYYQWC, (**B**) (Cyclo-1,6)CFSRMC, (**C**) (Cyclo-1,6)CLYHFC, (**D**) (Cyclo-1,6)CYTYWC, (**E**) (Cyclo-1,6)CLYKVC, and (**F**) RIF in the RpoB WT binding pocket as shown in the grey skeletal model. The blue area indicates the hydrophilic region, while the red area designates the hydrophobic region on the binding site.

To characterize the selected cyclic peptide ligands’ affinity towards the RpoB WT, these ligands were docked into the RIF binding site. The docking results showed that all ligands had suitable binding activity towards the RIF binding site of RpoB WT, with comparable but not better performance than RIF as the standard drug. An RMSD lower than 2 Å is often interpreted as an appropriate threshold for successful pose from a docking simulation [41]. Figure 3 visualizes the binding pose of each peptide and RIF on the RpoB WT docking site. 

**Figure 4:**
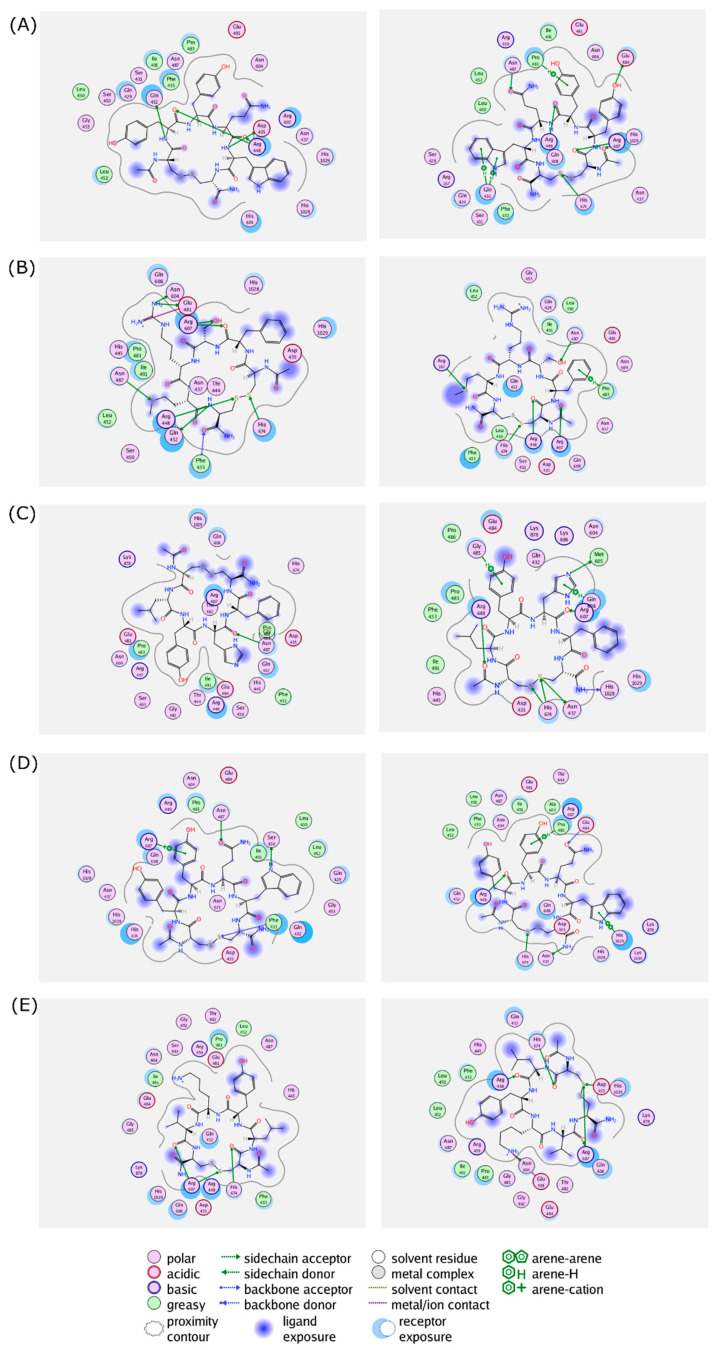
Protein–ligand interactions of (**A**) (Cyclo-1,6)CYYQWC, (**B**) (Cyclo-1,6)CFSRMC, (**C**) (Cyclo-1,6)CLYHFC, (**D**) (Cyclo-1,6)CYTYWC, and (**E**) (Cyclo-1,6)CLYKVC in the binding sites of RpoB WT (**left** panel) and S450L mutant (**right** panel) from a flexible molecular docking simulation. Hydrophobic and hydrophilic amino acid residues are represented in the green and purple circles, respectively. Interactions, such as hydrogen bond and arene–hydrogen, are represented by arrows, and ion contact is represented by purple dashed lines.

The 2D interaction between the cyclic peptides and the amino acid residues in the RpoB WT and S450L mutant are visualized and summarized in Figure 4 and Table S1, respectively.

#### 3.1.3. Docking to RpoB Mutants

To test the affinity of the selected cyclic peptides toward mutants of RpoB, these cyclic peptides and RIF were docked to RpoB S450W, RpoB H445Y, and RpoB D435V mutant structures with their docking scores and RMSDs on Table 2. Here, RIF showed structural incompatibility with the mutant receptor binding sites with RMSD values of 2.29 or higher. Some of the ligands were also predicted to have a poor interaction with certain RpoB mutant(s); for example, CFSRMC had RMSD values 2.48 and 2.57 against the S450W and H445Y mutants, respectively. Similarly, CYTYWC had an RMSD of 3.89 against the S450W mutant.

From the interaction analysis, it was shown that most of the cyclic peptides were able to interact with the binding pockets of RpoB mutants by forming hydrogen bonds or through hydrophobic interaction. The interacting amino acid residues and the binding modes were summarized in Table S2.

#### 3.1.4. Cyclization Effect

To analyze whether the cyclization has an impact on binding towards the receptor, the linear peptide structure of the selected cyclic peptide was generated and subjected to the identical preparation steps for the cyclic peptide compound library. Flexible docking simulations were carried out between the linear peptides and the RIF binding site of the RpoB, and the results were summarized in Table 3. Almost all of the linear peptides exhibited improperly high RMSD values during binding to RpoB WT and S450L, except during binding of linear-CLYHFC with RpoB S450L.

**Figure 5:**
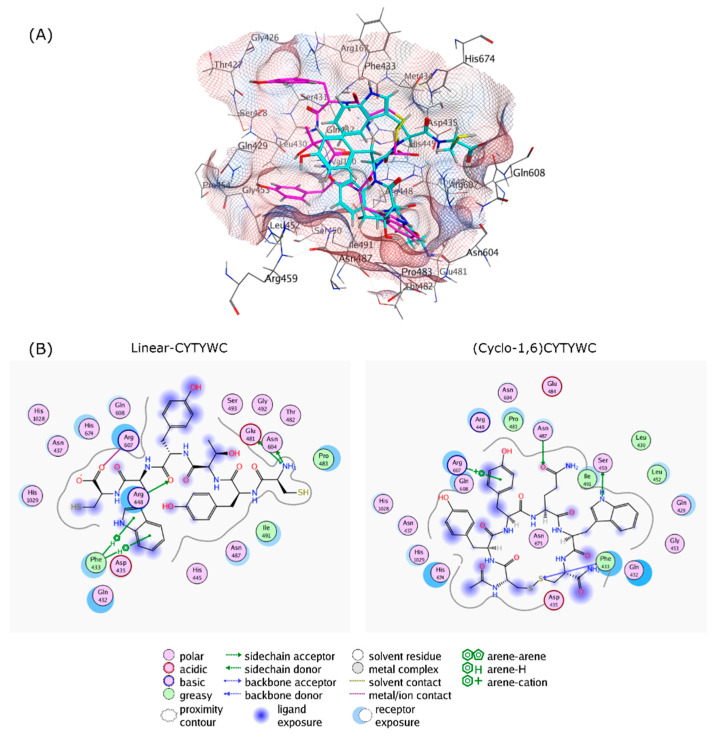
The cyclization effect, as follows: (**A**) 3D Protein–ligand interactions of linear-CYTYWC (cyan) and (Cyclo-1,6)CYTYWC (magenta), and (**B**) 2D interaction of linear-CYTYWC and (Cyclo-1,6)-CYTYWC with RIF binding site on RpoB. Hydrophobic and hydrophilic amino acids residues are represented in green and purple circles, respectively. Interactions, such as hydrogen bond and arene–hydrogen, are represented by arrows, and ion contact is represented by purple dashed lines.

As all of the peptides showed similar trends, the interactions of both linear and cyclic CYTYWC peptides and the RpoB WT binding site are visualized in Figure 5. Compared to the cyclic peptide, the linear peptide loses some hydrogen bonds, with lower hydrophobic contact to the binding site compared to the cyclic peptide.

#### 3.1.5. Computational Pharmacological Properties Prediction

The compatibility of ligands as an oral drug candidate can be characterized through several pharmacology parameters, such as physicochemical, pharmacokinetics (adsorption, distribution, metabolism, and excretion profile), and toxicity properties. Several online tools have been used to predict the physicochemical properties (Osiris DataWarrior), pharmacological properties (admetSAR, SwissADME, and pkCSM), and toxicological properties (ToxTree, admetSAR) of the selected ligand to screen its suitability for drug development during the early stage of drug discovery. Table 4 summarizes the physicochemical properties of selected cyclic peptide ligands and RIF, such as molecular weight, octanol–water partition coefficient (logP), total H-donor and H-acceptor, and total polar surface area (TPSA).

The computational prediction of the ADME properties were summarized in Table 5. Absorption properties were represented by the ability of the ligand to interact with a P-glycoprotein (P-gp) efflux transporter either as a substrate or as an inhibitor. Unlike RIF, the cyclic peptides could not act as a P-gp inhibitor. All cyclic peptide ligands were predicted not to interact with CYP450, either as a substrate or as an inhibitor. Total clearance was used as the descriptor for excretion. From the result, negative value for this descriptor predicts the tendency of these compounds to be accumulated in the body by avoiding rapid renal clearance or proteolysis.

A QSAR-based decision tree approach was used to predict the ligand toxicological properties [35,42]. Table 6 summarizes the predicted toxicological properties of selected ligands and RIF based on the Benigni–Bossa rule base for mutagenicity and carcinogenicity [43]. Structural alerts described the functional group/moiety that is linked to the carcinogenic activity of chemicals and extrapolated from the structure of major carcinogen chemical classes. From the result, only CLYHFC has a structural alert for nongenotoxic carcinogenicity, which is due to the imidazole ring of the histidine residue. Selected peptide ligands and RIF were not identified as hERG potassium ion channel inhibitors.

#### 3.1.6. N-Terminus and C-Terminus Modification

Structural modification through N-terminus acetylation and C-terminus amidation were carried out to improve the plasma stability and bioactivity of the synthetic peptides. To observe whether the modification will alter its binding to the docking site, the modified peptide structures were generated and docked to RpoB WT and its mutants. The result is summarized in Table 7 and all of the modified peptides exhibited proper RMSD values. The cyclic peptide modification did not reduce its binding affinity to the receptor compared to the parent peptide. 

### 3.2. Solid-Phase Peptide Synthesis

**Figure 6:**
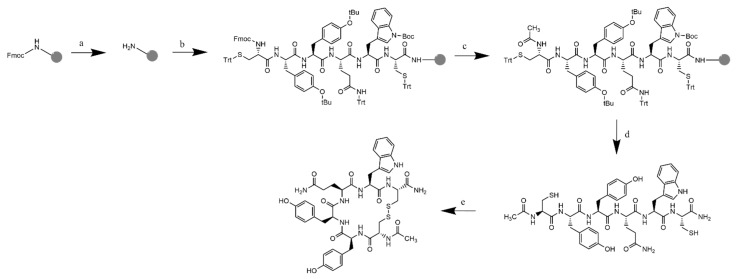
Synthetic scheme of (Cyclo-1,6)Ac-CYYQWC-NH_2_ with Rink amide MBHA resin: (**a**) Fmoc deprotection: piperidine 20% in DMF; (**b**) addition of Fmoc-L-Cys(Trt)-OH and Fmoc-L-Trp(Boc)-OH. Fmoc-L-Gln(Trt)-OH, Fmoc-L-Tyr(tBu)-OH (2×), Fmoc-L-Cys(Trt)-OH with HCTU/NMM in DMF as the coupling reagent; (**c**) final deprotection with 20% piperidine in DMF, followed by acetylation to the N-terminus with acetic anhydride and 2.6-lutidine in DMF, RT; (**d**) resin cleavage and global deprotection: TFA/TIPS/Phenol/H_2_O (90:5:2.5:2.5), RT, 2 h reaction, followed by crude precipitation and RP-HPLC purification resulted in the linear-Ac-CYYQWC-NH_2_; and (**e**) cyclization with air dilution in a high dilution of ammonium bicarbonate buffer pH = 8.5, followed by characterization.

The modified peptides were synthesized using solid-phase peptide synthesis with Fmoc chemistry according to the synthetic strategy depicted in Figure 6. Rink amide resin with a MBHA linker (2.5 mmol loading) was used as the solid support to synthesize the C-amidated linear hexapeptides. After the last cycle of deprotection of the Fmoc group with piperidine, the resin was washed, and the N-terminus was acetylated with acetic anhydride. Cleavage from the resin was successfully performed with the cleavage cocktail of trifluoroacetic acid, saturated phenol, water, and triisopropylsilane (90:5:2.5:2.5, 30 mL) for 2 h. The peptide was separated from the non-peptide components, such as the protecting groups, through precipitation with cold ether. Purification of the crude peptides with semi-preparative HPLC with a C-18 column and UV detector (ʎ = 214.4 nm) resulted in pure fractions containing the linear peptides; they were characterized with ESI mass spectrometry before undergoing the cyclization process.

After HPLC analysis of each fraction of the linear peptide, the fractions containing pure peptide were pooled and concentrated. Then, the concentrate was dissolved in 1 L of ammonium bicarbonate buffer at pH = 8.5 as a highly diluted solution. The solution was subjected to air oxidation with stirring for 24 h to form an intramolecular disulfide bond between two sulfhydryl moieties on the two cysteine residues. The dilute peptide solution is necessary to maintain low intermolecular interactions between the linear peptide that can be resulted in cyclic dimer, trimer, or oligomer formation. After the cyclization reaction was completed, the solvent was removed, and the resulting cyclic peptides were subjected to semi-preparative HPLC to remove cyclic dimers, trimers, and buffer salts. The pure fractions were pooled and lyophilized. Analytical HPLC was also carried out to identify the purity of the cyclization product. The pure cyclic peptide was characterized with mass spectrometry to confirm the disulfide bond formation. From the mass spectra, it was observed that cyclization resulted in the product with molecular weight difference of 2 Da (Figure S1), respective to the loss of two hydrogen atoms during cysteine oxidation to cystine.

For all five cyclic peptides, a peak shifting was observed in the cyclic peptide chromatogram compared to the linear peptide chromatogram. The cyclic peptides were eluted from the reversed-phase column faster than the linear counterparts, indicating increasing of polarity of the cyclization product (Figure S2).

As the summary, the exact mass spectra of the synthetic linear and cyclic peptides were summarized in Table S3. All of the peptides were detected with the LR-MS with 100% intensity as [M+H]^+^ ions. From calculation, it is observed that the exact mass is almost identical to the calculated monoisotopic mass, with the maximum deviation of 0.03%. Another characterization was carried out with ^1^H NMR with DMSO-d6 as the solvent, and the proton NMR spectra of each cyclic peptides were visualized in Figure S3.

## 4. Discussion

Identification of a novel inhibitor targeting transcription process in MTB is critical in addressing the RIF resistance problem. In this paper, we report structural design and synthesis of cyclic peptides as potential inhibitors of MTB RNAP. We conducted computational design and screening to retrieve the selected peptide sequences with a potential binding affinity to the RIF binding site in RpoB WT and mutants. The pharmacological properties were predicted using several online tools to assess the drug-likeness of the cyclic peptide drug candidates. Afterwards, we examined the drug candidates with a series of computational studies to observe the cyclization effect. To address the absorption profile, structural modification with the N-terminus acetylation and C-terminus amidation approach were carried out on the selected cyclic peptides. We employed solid-phase peptide synthesis to produce the desired modified cyclic peptides, to be tested in the next phase of this study. These cyclic peptides were expected to inhibit the MTB transcription initiation complex with a similar mode of action as RIF, blocking the nucleotide addition site by steric clash and inducing abortive initiation. Initial studies showed that (Cyclo-1,6)Ac-CLYHFC-NH_2_ had potential activity to inhibit RNA elongation in the MTB transcription initiation assay in the nanomolar range with RIF as a positive control. In addition, (Cyclo-1,6)Ac-CYTYWC-NH_2_ had biological activity potential in the micromolar range. However, the other three cyclic peptides and all of the linear peptides had no activity in inhibiting RNA elongation in MTB transcription initiation assay. Detailed studies of their biological activities will be presented in the future.

A library containing 7500 cyclic hexapeptides ligands was generated for the purpose of this study. The designed ligand has cysteines at both of its termini to facilitate the formation of disulfide bridge for the cyclization. The ligands were designed as hexapeptides to produce molecules with similar average size and weight to RIF. The RIF binding site on RpoB was used as the docking site to find the best binding pose of the ligands. This cyclic peptide library was subjected to a series of computational screenings with rigid docking and flexible docking simulations. From the flexible docking simulation, we have chosen five cyclic peptide sequences that could bind to the RIF binding site in the RpoB WT and S450L mutant. These selected cyclic peptides returned a good docking score compared to RIF, and also returned appropriate RMSD values. All of the cyclic peptides appeared to interact with residues Phe433, Arg448, and His674.

Typically, RIF resistance is associated with the mutation that occurs within the RRDR. Among the mutations that occur, S450L mutation is commonly found in clinical isolates of MDR-RR TB patients. Substitution of serine to leucine involves change in one nucleotide (TCG to TTG) and altered the characteristics RIF binding site of the mutant as the consequence. This change causes RIF resistance from the lack of van der Waals interaction and loss of the free binding energy [44]. From the 2D interaction analysis, it was observed that most of the cyclic peptides could interact with the serine or leucine residue on the 450 positions (S531L in *E. coli* RpoB consensus numbering).

Additionally, to test whether the selected peptides are capable of forming an interaction with other altered RIF binding sites on DR-TB associated mutants, docking studies were carried out between the ligands and selected mutants of RpoB (S450W, H445Y, and D435V). The results suggest that most of the selected peptides might be able to bind to some other RNAP variants; thus, they highlighted the potential of these compounds as potential therapeutics for DR-TB. While all of the peptides exhibited a high binding affinity to the RpoB WT, some of the cyclic peptides could also interact with the W450 residue on interaction with the S450W mutant, and with the V435 residue on interaction with the D435V mutant. In contrast, RIF did not bind favorably to RpoB mutants.

To provide insight on the cyclization effect, a docking simulation was performed on the linear structure of the selected peptides. Docking results showed that the linear peptide exhibited weaker interactions with the RIF binding site, caused by the loss of hydrogen bonding and less hydrophobic interaction (shown as example in Figure 4). This could be explained by the higher degree of flexibility of the linear peptide, while cyclization provides conformational restriction and more often leads to higher binding selectivity [45].

Computating the pharmacological properties prediction was carried out with the aid of several online tools to calculate the physicochemical properties and to predict the ADME-Tox properties. From the result, it was observed that almost all ligands, including RIF, violate Lipinski’s ‘rule of five’ for oral drugs (molecular weight less than 500 Da, having no more than 5 hydrogen bond donors and 10 hydrogen bond receptors, and a calculated logP more than 5) [46]. However, it is worth mentioning that RIF is mostly administered as an oral drug to the patients, despite the molecular weight and the number of hydrogen bond donors and acceptors; this is presumably due to its cLogP at 4.71. Indeed, RIF is known to be readily absorbed from the GI tract to the plasma, reaching the maximum plasma concentration after 1 to 3 h from administration of a 10 mg/kg dose [47,48]. 

The P-gp interaction reflects the plasma and tissue concentration in human body after the administration of a foreign substance, and is often used as the parameter to predict the absorption profile of drug candidate [49,50]. From the results, all peptide ligands are predicted to be a P-gp substrate, while RIF could act as both a substrate and inhibitor against this transporter. These data indicate the possibility of reduced serum concentrations after cyclic peptides administrations. In contrast, RIF has the chance to be transported across cell membranes into the cell. Furthermore, RIF is a known potent inducer of cytochrome P-450 (CYP450), and drug–drug interactions are often found in TB patients on treatment due to this fact [51,52,53]. The drug distribution is represented by a fraction unbound which describe the ratio of the free drug (not bound to plasma) proportional to the total concentration absorbed. It was observed that RIF had the lowest fraction unbound compared to the cyclic peptides, and this corresponded to a previous study on RIF–plasma protein binding. More than 80% of the concentration of RIF will be retained in the plasma; thus, it limits the amount of RIF that penetrates the tissue which reaches the drug target [54,55]. This means that a higher dose is needed to facilitate the distribution of the drug to the drug target. However, decreased plasma binding would also cause a shorter half-life for the compound, and plasma binding could aid with the prolonged duration of action. Therefore, further studies are needed to determine the plasma binding equilibrium condition for the development of our cyclic peptides. A distribution profile was predicted with the total clearance parameter, in which the results showed that RIF and the cyclic peptides tend to be accumulated in the body. Although it is hypothesized that cyclization could prolong the half-lives of the peptide, this result has yet to be validated with an elimination study, as small peptides with a molecular weight of less than 2 kDa are prone to rapid renal clearance [29,56].

A SAR-based prediction was used to predict the toxicity properties of the selected ligands and RIF, using the open-source ToxTree software. From the prediction result, (Cyclo-1,6)CLYHFC has a structural alert for nongenotoxic carcinogenicity from the imidazole ring in the histidine side chain. Although the SAR-based prediction linked the imidazole moiety to the 4-methylimidazole carcinogenicity, histidine is known to be a non-carcinogen after both long-term and short-term exposure and, instead, histidine can boost methotrexate effectiveness in cancer treatment [57,58]. Other peptides were not found to be a potential mutagen or carcinogen.

Two approaches, namely N-terminus acetylation and C-terminus amidation, can be used to improve the bioavailability of the bioactive peptides. This approach reduces the overall charges of the peptides, leading to better stability and absorption, and also avoiding the enzymatic degradation to both of the termini caused by various peptidases [59,60,61]. However, this modification could also alter the protein–ligand binding affinity. To assess whether modification would eliminate the binding affinity of the selected peptides, we generated the modified cyclic peptides structures and subjected them to the flexible docking simulation. The results showed that these modifications did not eliminate the binding affinity and, in some cases, modification resulted in improved docking scores.

After the computational study and examination, we proceeded to synthesize the modified peptides. Synthesis of the modified peptides were conducted through solid-phase peptide synthesis with Fmoc chemistry on a Rink amide resin with a MBHA linker to produce an amidated C-terminus. The synthesis produced the expected yield of linear peptides with reasonable purity. The presence of appropriate scavengers (i.e., TIPS, saturated phenol, and water) successfully prevented side reactions, such as peptide alkylation, due to the release of active carbocations from the side chain protecting groups (i.e., Trityl and t-Butyl) during the TFA cleavage procedure. Purifications with a semi-preparative reversed-phase HPLC with a C18 column were able to produce linear peptides with a purity higher than 98%, as characterized by analytical HPLC and ESI-MS. The cyclization reaction produced a high yield of cyclic monomer, and the cyclic monomer was separated by semi-preparative HPLC from the very low amounts of cyclic dimer, trimer, and tetramers that were formed during the cyclization reaction in a highly diluted condition. During cyclization reactions, we also found a low amount of oxidation products in peptides that contained Met, Trp, and Tyr residues. Several common side oxidation products were observed during the cyclization of (Cyclo-1,6)Ac-CYYQWC-NH_2_ and (Cyclo1,6)Ac-CYTYWC-NH_2_, which were due to the oxidation of Trp or Tyr residue with the addition of 17 Da to the peptide molecular weights. Similarly, the oxidation product of (Cyclo-1,6)Ac-CFSRMC-NH_2_ was also found by the formation of a sulfoxide group on the Met residue of the peptide.

## 5. Conclusions

A cyclic peptide library was constructed based on the RIF binding site of RpoB. The library was subjected to tiered computational screenings with molecular docking simulations and resulted in five cyclic peptides with a high binding affinity and proper RMSD values during interactions with RpoB WT and S450L mutants. We computationally assessed peptide binding properties with other RIF resistant associated mutants, the cyclization effect, and the terminus modification effects on the peptides, and the predicted pharmacological properties of the selected cyclic peptides. The resulting cyclic peptides were successfully synthesized and purified to give the desired cyclic peptides with high purities, as determined by HPLC, the LC-ESI-MS^+^ system, and proton NMR. As with RIF, these peptides are being fully evaluated for their biological activities to inhibit RNA elongation in the MTB transcription initiation assay.

## Figures and Tables

**Table 1:** Flexible docking scores of selected peptide ligands to RpoB WT and S450L.

Peptide Sequence	RpoB S450L		RpoB WT	
	Docking Score	RMSD (Å)	Docking Score	RMSD (Å)
(Cyclo-1,6)CYYQWC	−13.8389	1.3834	−12.5909	1.7077
(Cyclo-1,6)CFSRMC	−12.7736	1.9083	−12.9260	1.8025
(Cyclo-1,6)CLYHFC	−12.6189	0.7636	−11.5646	1.4826
(Cyclo-1,6)CYTYWC	−12.4056	1.2551	−13.5010	1.5387
(Cyclo-1,6)CLYKVC	−12.1895	1.6815	−11.9209	1.7620
RIF	−12.0894	7.8294	−13.9747	0.7992

**Table 2:** Flexible docking scores of selected cyclic peptide ligands to other RpoB mutants.

Peptide Sequence	RpoB S450W		RpoB H445Y		RpoB D435V	
	Docking Score	RMSD (Å)	Docking Score	RMSD (Å)	Docking Score	RMSD (Å)
(Cyclo-1,6)CYYQWC	−11.5118	1.5326	−12.4968	1.5917	−13.4142	1.5039
(Cyclo-1,6)CFSRMC	−12.9039	2.4778	−12.4967	2.5737	−12.2775	1.6661
(Cyclo-1,6)CLYHFC	−12.0156	1.8121	−11.4236	1.5163	−11.9727	1.9666
(Cyclo-1,6)CYTYWC	−11.9482	3.8997	−12.7385	1.7487	−13.7298	1.7003
(Cyclo-1,6)CLYKVC	−12.2447	1.4748	−12.0770	1.1750	−11.2436	1.6141
RIF	−11.6767	10.6001	−12.5410	9.7588	−12.2798	2.2929

**Table 3:** Flexible docking scores of linear peptides to RpoB WT and S531L.

Peptide Sequence	RpoB S450L		RpoB WT	
	Docking Score	RMSD (Å)	Docking Score	RMSD (Å)
Linear-CYYQWC	−13.5083	4.4259	−12.8980	2.2476
Linear-CFSRMC	−12.0515	2.9071	−12.4066	2.4019
Linear-CLYHFC	−13.6569	1.5650	−12.9947	2.4327
Linear-CYTYWC	−12.9043	3.0690	−12.2946	3.7024
Linear-CLYKVC	−12.8009	2.0484	−12.4622	3.1910

**Table 4:** Physicochemical properties of selected ligands.

Peptide Sequence	Weight (Da)	ClogP	H-Donor	H-Acceptor	TPSA (Å^2^)
(Cyclo-1,6)CYYQWC	862.98	−2.3098	10	18	363.21
(Cyclo-1,6)CFSRMC	744.94	−4.5460	10	17	373.04
(Cyclo-1,6)CLYHFC	782.94	−2.0673	8	16	312.78
(Cyclo-1,6)CYTYWC	835.96	−2.1119	10	17	340.35
(Cyclo-1,6)CLYKVC	726.94	−1.8108	8	15	311.74
RIF	822.41	4.7129	6	16	220.15

**Table 5:** Computational ADME properties prediction of selected ligands.

Peptide Sequence	Absorption		Distribution	Metabolism		Excretion
	P-gp Substrate	P-gp Inhibitor	Fraction Unbound	CYP450 Substrate	CYP450 Inhibitor	Total Clearance
(Cyclo-1,6)CYYQWC	Yes	No	0.280	No	No	−0.605
(Cyclo-1,6)CFSRMC	Yes	No	0.590	No	No	−0.097
(Cyclo-1,6)CLYHFC	Yes	No	0.379	No	No	−0.650
(Cyclo-1,6)CYTYWC	Yes	No	0.260	No	No	−0.656
(Cyclo-1,6)CLYKVC	Yes	No	0.464	No	No	−0.432
RIF	Yes	Yes	0.120	3A4	No	−0.558

**Table 6:** Computational toxicological properties prediction of selected ligands.

Peptide Sequence	Structural Alert for Genotoxic Carcinogenicity	Structural Alert for Nongenotoxic Carcinogenicity	Potential *S. thypimurium* TA100 Mutagen Based on QSAR	Potential Carcinogenicity Based on QSAR	hERG Inhibitor
(Cyclo-1,6)CYYQWC	No	No	No	No	No
(Cyclo-1,6)CFSRMC	No	No	No	No	No
(Cyclo-1,6)CLYHFC	No	Yes	No	No	No
(Cyclo-1,6)CYTYWC	No	No	No	No	No
(Cyclo-1,6)CLYKVC	No	No	No	No	No
RIF	Yes	No	No	No	No

**Table 7:** Flexible docking score of modified cyclic peptides to RpoB WT and S450L.

Peptide Sequence	RpoB S450L		RpoB WT	
	Docking Score	RMSD (Å)	Docking Score	RMSD (Å)
(Cyclo-1,6)Ac-CYYQWC-NH_2_	−13.4662	1.6298	−13.5887	1.5582
(Cyclo-1,6)Ac-CFSRMC-NH_2_	−11.5095	1.4119	−12.0499	1.2679
(Cyclo-1,6)Ac-CLYHFC-NH_2_	−12.1731	1.0797	−11.3917	1.9142
(Cyclo-1,6)Ac-CYTYWC-NH_2_	−13.1416	1.6129	−12.6561	1.8910
(Cyclo-1,6)Ac-CLYKVC-NH_2_	−11.7044	1.7218	−11.5233	1.0785

## Supplementary Materials

The following supporting information can be downloaded at: https://www.mdpi.com/article/10.3390/life12091333/s1, Figure S1: Mass spectra of synthetic peptides, Figure S2: HPLC chromatograms of synthetic peptides; Figure S3: Proton NMR spectra of cyclic peptides; Table S1: Interacting amino acid residues: Docking to RpoB S450L and WT; Table S2: Interacting amino acid residues: Docking to RpoB mutants; Table S3: Exact mass of synthetic peptides.
