# Supplementary material for: Structural Design and Synthesis of Novel Cyclic Peptide Inhibitors Targeting Mycobacterium tuberculosis Transcription
Source: Life (Basel). 2022 Aug 28;12(9):1333. doi: 10.3390/life12091333 (PMC9506182; doi:10.3390/life12091333)
Supplement: Supplementary file 1 [file life-12-01333-s001.zip › life-1876772-supplementary.pdf]

Table S1. Docking to RpoB S450L and WT: Interacting Amino Acid Residues

Peptide Sequence	RpoB WT	RpoB S531L/S450L MTB
(Cyclo)CYYQWC	**Asn604, Arg607, Arg448, Asp435, Phe433,** Glu481, Thr482, Pro483, Gln608, His1028, His1029, Asn437, His674, Ile491, Gln432, Asn487, Leu452, Gln429, Gly453, Arg459, Ser450	**Asn604, Glu484, Arg448, Asp435, Asn437, Phe433, Gln429,** Glu481, Thr482, Gln608, Pro483, His1029, Arg607, His1028, His674, His445, Gln432, Gly453, Leu452, Leu450, Asn487, Ile491, Arg459
(Cyclo)CLYHFC	**Pro483, Glu484, Arg448, Arg607, Asn437, His674,** Asn604, Ile491, Gln608, His1029, His1028, Phe433, His445, Gln432, Asp435	**Arg448, Gln432, His674, Arg607, Gln608,** Glu481, Pro483, Glu484, Asn604, His1029, Asp435, His1028, Lys1034, Asn437, His445, Phe433, Leu450, Ile491
(Cyclo)CLYKVC	**Phe433, Lys886,** Gln432, Ile491, Asp435, His445, Arg607, Glu481, Ser493, Gly492, Thr482, Pro483, Arg448, His1028, Gly485, Asn604, Glu484, Met605, Met601, Lys878, His674, Asn437, His1029, Gln608	**Gln432, Phe433, His674, Asp435, Arg448, Arg607, Asn604, Gly481,** Ser431, Ser428, Arg176, Leu452, Leu450, Pro483, Asn487, Ile491, Thr444, Gly492, Ser493, Gln608, Asn437, Met434
(Cyclo)CFSRMC	**Gln432, Phe433, His674, Asp435, Arg607, Asn604, Glu481,** Arg167, Asn487, Ser450, Leu452, Ile491, Pro483, Thr444, Ser493, His445, Gln608, Met434, Gln429, Ser431, Ser428	**Gln432, Phe433, His674, Asp435, Arg448, Arg607, Asn604, Glu481,** Ser428, Ser431, Arg167, Leu452, Leu450, Pro483, Asn487, Ile491, Thr444, Gly492, Ser493, Gln608, Asn437, Met434, Phe433, His445
(Cyclo)CYTYWC	**Pro483, Arg448, Phe433, Arg167, His674,** Ile491, Asn487, Ser450, Gly453, Leu452, Gly426, Thr427, Leu430, Gln432, His445, Asp435, Asn604, Gln429, Ser431	**Gln432, Arg167, Arg448, Arg607, Pro483, Phe433, His674,** Leu450, Ser431, Leu452, Gln429, Thr427, Arg459, Gly426, Asn487, Ile491, His445, Asp435, Asn604, Ser428

*Amino acid residues written in **bold** interact with the binding pocket by forming hydrogen bonds/ arene-H interactions

Table S2. Docking to RpoB Mutants: Interacting Amino Acid Residues

**Peptide Sequence**	**RpoB S531W/S450W MTB**	**RpoB H526Y/ H445Y MTB**	**RpoB D516V/ D435V MTB**
(Cyclo)CYYQWC	**Asn604, Arg607, Phe433, Arg448, Asp435,** Ile491, Pro483, Glu481, Glu484, Thr482, His1028, Gln608, His1029, Asn437, His674, Gln432, Asn487, Trp450, Arg459	**Phe433, Asn604, Arg448, Asp435**, Glu481, Glu484, Pro483, Thr482, His1029, Gln608, His1028, Asn437, His674, Ile491, Gln432, Leu452, Gln429, Gly453, Ser450, Asn487	**Asn604, Phe433, Arg448, Asn437,** Glu484, Glu481, Thr482, Pro483, Arg607, Gln608, Ile491, Asn487, Ser450, His1029, His1028, His674, Val435, Gln432, Leu430, Leu452, Gln429, Gly453
(Cyclo)CFSRMC	**Pro483, Glu484, Glu481, Asn604, Asn437, Asp435,** Thr444, Trp450, Ile491, Gln432, His445, Phe433, His674, Arg448, His1029, Gln608, His1028, Ser493	**Gln432, Phe433, His674, Asp435, Arg448, Arg607, Asn604, Glu481,** His1029, Gln608, Ser493, Thr444, Pro483, Ile491, Asn487, Leu452, Ser428, Arg167, Ser431, Gln429, Met434	**Gln432, Phe433, His674, Arg448, Glu481, Gln608, Arg607**, Arg167, Asn487, Leu452, Ser450, Ile491, Pro483, Thr444, Thr482, Ser493, Gly492, Asn604, His1029, Val435, Ser431, Gln429, Ser428
(Cyclo)CLYHFC	**Asn604, Glu484, Arg607, His674, Phe433, Asn437,** Glu481, Pro483, Ile491, Arg448, Trp450, Gln432, His445, His1028, Asp435, Gln608, His1029	**Pro483, Arg448, Glu484, His674, Asn437, Arg607,** Glu481, Asn604, Ile491, Gln608, His1029, His1028, Phe433, Gln432, Asp435	**Arg448, Gln432, Ser450, Arg607, Gln608, Phe433,** Glu484, Pro486, Gly485, Asn487, Pro483, Asn604, Ser493, Thr444, Ile491, Gly492, Glu481, Val170, Val435, His674, Asn437

(Cyclo)CYTYWC	**Glu484, His1029, Asn437, His674, Asp435, Phe433,** Thr482, Pro483, Met605, Trp450, Asn604, Glu481, Ile491, Lys886, Met601, Lys878, Gln608, Arg607, His445, Arg448, Gln432, Ser431, Asn487	**Arg607, Asp435, Arg448, His674, Phe433, Pro483, Arg167,** Ile491, Asn487, Ser450, Gly453, Leu452, Gly426, Thr427, Ser431, Gln432, Tyr445, Gln429, Leu430, Asn604	**Gln432, His674, Phe434, Arg607, Arg448,** Ser450, Pro483, Ile491, Asn487, Gly453, Leu452, Asn604, Gln428, Ser431, Leu430, Val435, Ser428, His445, Thr427, Arg167
(Cyclo)CLYKVC	**Phe433, Lys886,** Ser493, Thr482, Gly492, Pro483, Ile491, Glu481, Asp435, Arg607, Gln432, His445, His674, Arg448, Asn437, His1029, His1028, Lys878, Met601, Met605, Glu484, Gly485, Asn604	**Arg607, Lys886, Phe433,** Asn437, His674, Arg448, Ile491, Gln432, Gly492, Ser493, Pro483, Glu481, Gln608, Asp435, Thr482, Gly485, Glu484, Asn604, Met601, His1028, Lys878, Met605	**Lys886,** Asn437, Val435, Phe433, Thr482, Gln432, Arg448, Glu481, Ile491, Ser493, Gly492, Pro483, Gly485, Asn604, Glu484, Met605, His1028, Arg607, Met601, Lys878, Gln608, His1029

*Amino acid residues written in **bold** interact with the binding pocket by forming hydrogen bonds/ arene-H interactions

Figure S1. Mass spectra of linear and cyclic peptides: (A) Ac-CYYQWC-NH_2, (B) Ac-CFSRMC-NH_2, (C) Ac-CLYHFC-NH_2, (D) Ac-CYTYWC-NH_2, and (E) Ac-CLYKVC-NH_2. Mass spectra for the linear and cyclic peptides were shown on the left and right side, respectively. All mass spectra were obtained with TOF MS ES+ with C-18 column, polar solvent = 0.08% formic acid and non-polar solvent = 100% acetonitrile.

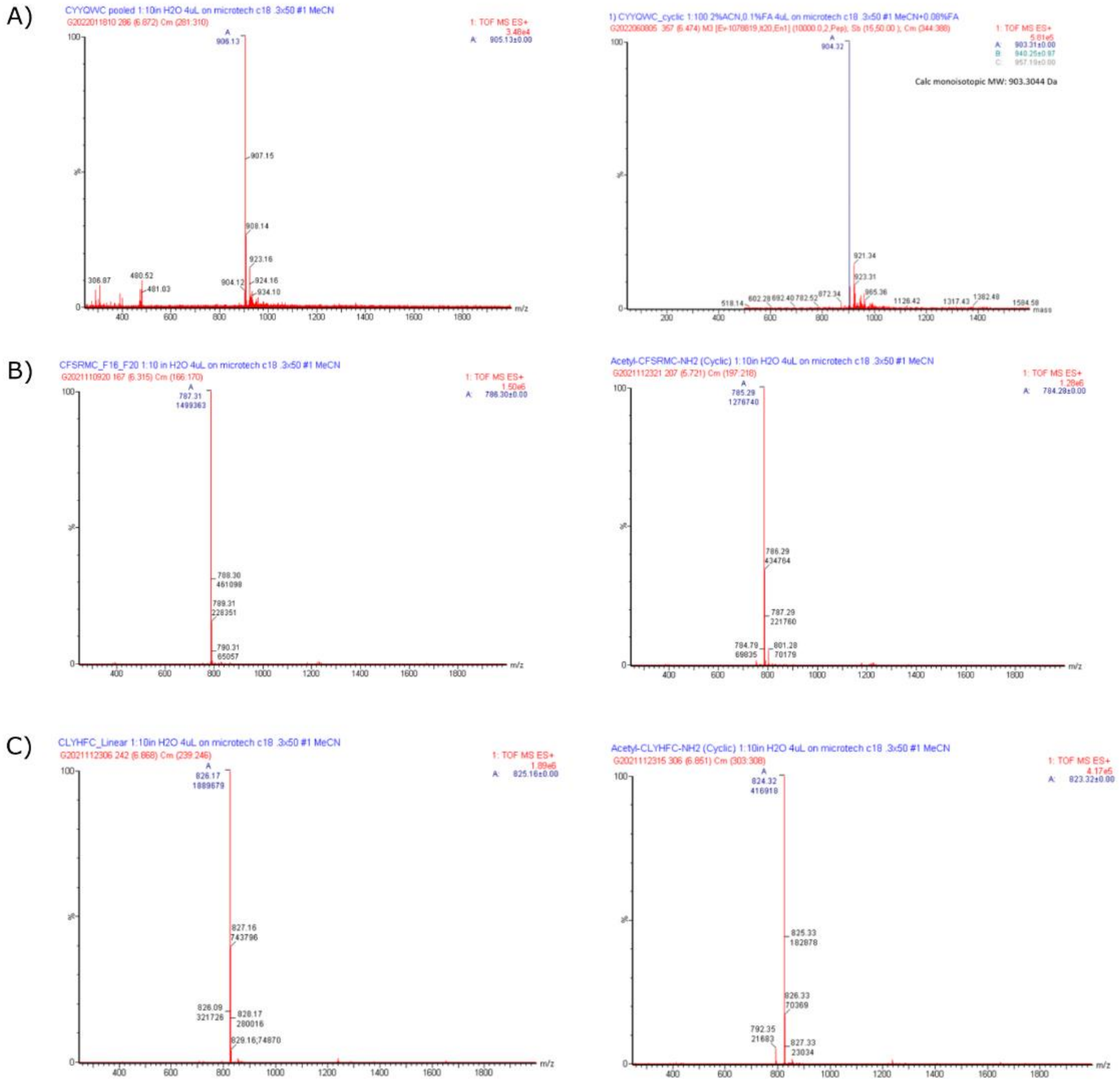

D)

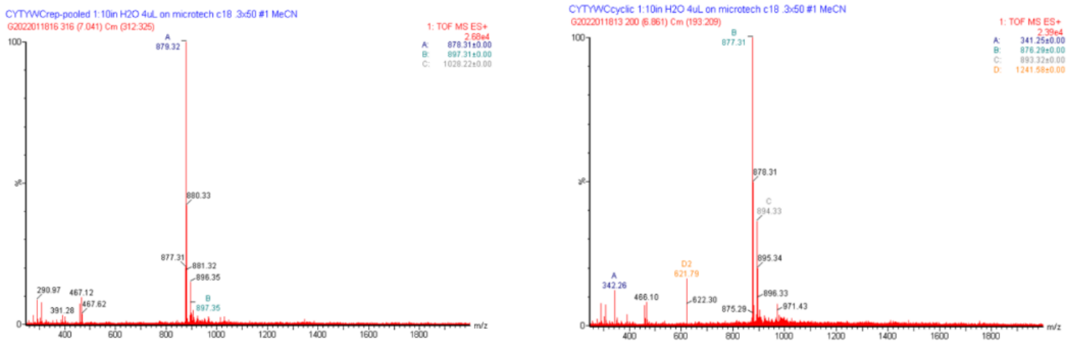
CYTYWCrep-pooled 1:10in H2O 4uL on microtech c18 .3x50 #1 MeCN
G2022011816 316 (7.041) Cm (312:325)
1: TOF MS ES+
2.68e4
A: 878.31±0.00
B: 897.31±0.00
C: 1028.22±0.00
879.32
880.33
877.31
881.32
896.35
897.35
290.97
467.12
391.28
467.62
m/z
CYTYWCcyclic 1:10in H2O 4uL on microtech c18 .3x50 #1 MeCN
G2022011813 200 (6.861) Cm (193:209)
1: TOF MS ES+
2.39e4
A: 341.25±0.00
B: 876.29±0.00
C: 893.32±0.00
D: 1241.58±0.00
877.31
878.31
894.33
895.34
342.26
621.79
466.10
622.30
875.29
896.33
971.43
m/z

E)

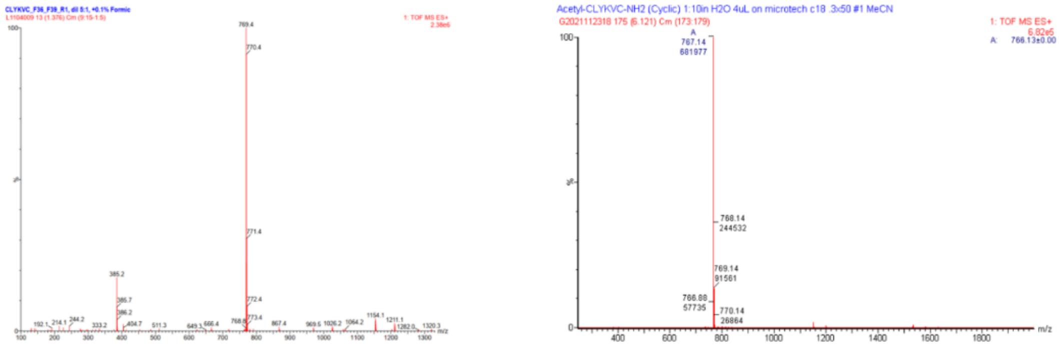
1: TOF MS ES+
769.4
770.4
771.4
772.4
773.4
385.2
Acetyl-CLYKVC-NH2 (Cyclic) 1:10in H2O 4uL on microtech c18 .3x50 #1 MeCN
G2021112318 175 (6.121) Cm (173:179)
1: TOF MS ES+
6.82e5
A: 766.13±0.00
767.14
681977
768.14
244532
769.14
91561
766.88
57735
770.14
26864
m/z

Figure S2. HPLC analysis of linear and cyclic peptides: (A) Ac-CYYQWC-NH_2, (B) Ac-CFSRMC-NH_2, (C) Ac-CLYHFC-NH_2, (D) Ac-CYTYWC-NH_2, and (E) Ac-CLYKVC-NH_2. Chromatograms for the linear and cyclic peptides were shown on the left and right side, respectively. Cyclic peptides were eluted from the column with faster retention time than the linear form. All chromatograms were obtained with C-18 column, with polar solvent 94.9% water, 5% acetonitrile, and 0.1% TFA, and nonpolar solvent 100% acetonitrile.

(A) Ac-CYYQWC-NH_2

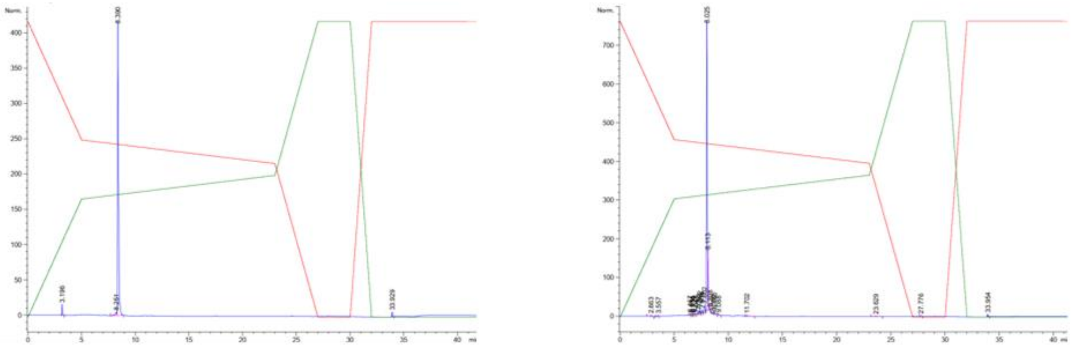

(B) Ac-CFSRMC-NH_2

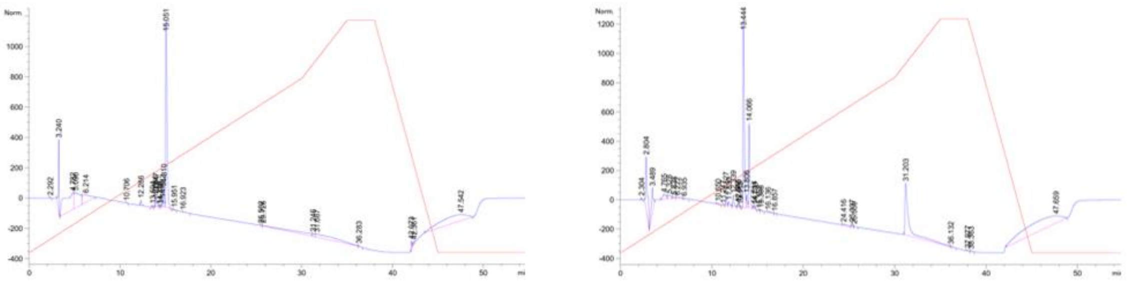

(C) Ac-CLYHFC-NH_2

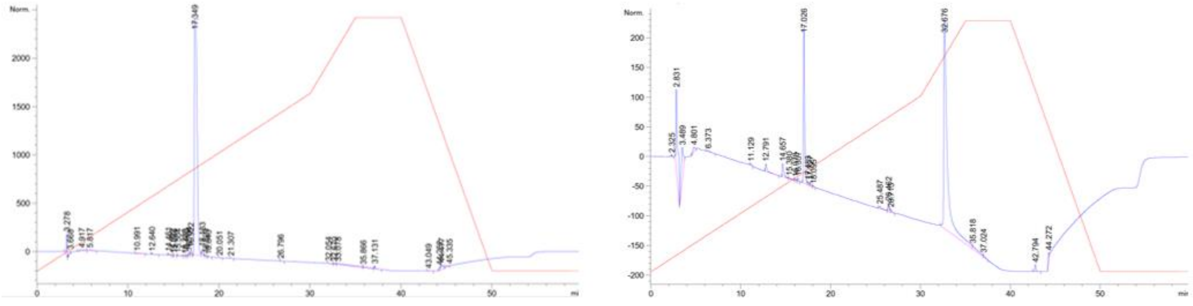

(D) Ac-CYTYWC-NH_2

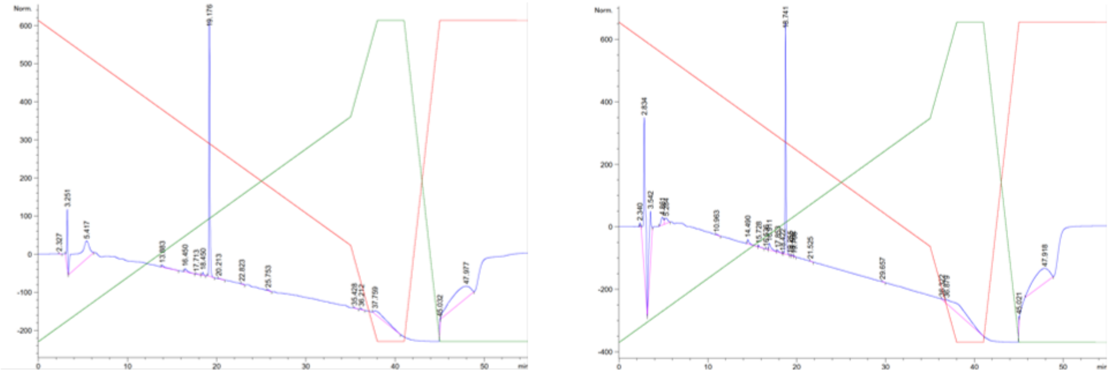

(E) Ac-CLYKVC-NH_2

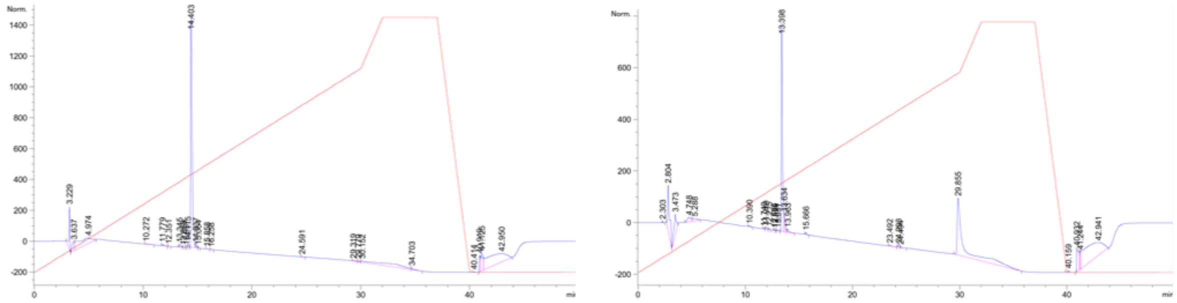

Figure S3. Proton NMR Spectra: Cyclic peptides (500 MHz, DMSO-d6)

A. (Cyclo)Ac-CYYQWC-NH_2

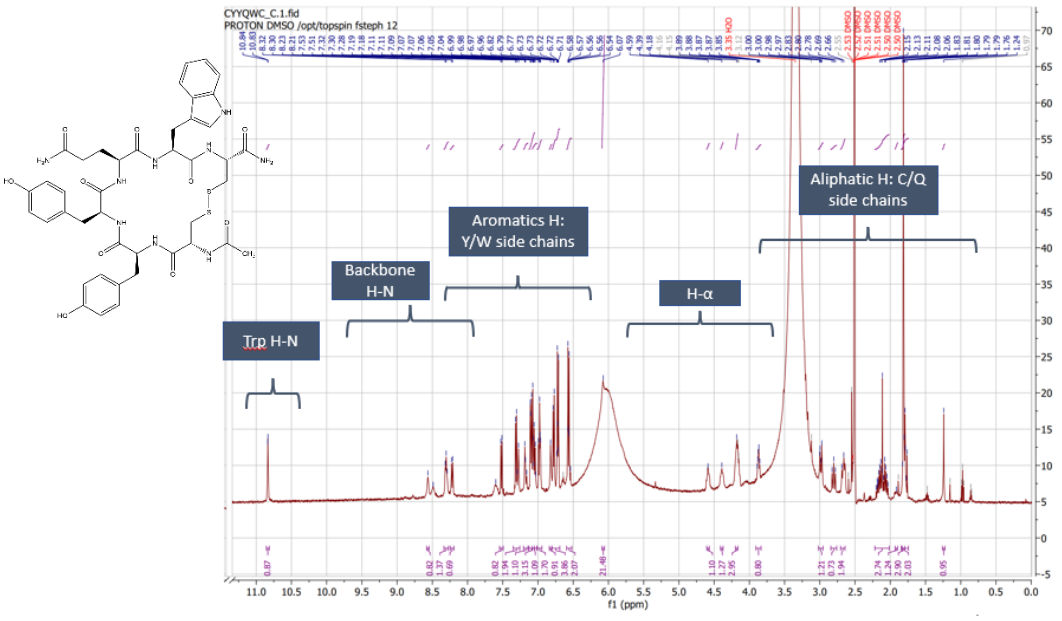

^{1}H NMR (500 MHz, DMSO) δ 10.84 (d, J = 2.4 Hz, 1H), 8.56 (s, 1H), 8.30 (t, J = 7.1 Hz, 1H), 8.22 (d, J = 8.7 Hz, 1H), 7.52 (d, J = 7.9 Hz, 1H), 7.35 – 7.26 (m, 2H), 7.20 – 7.14 (m, 1H), 7.13 – 7.05 (m, 3H), 7.08 – 7.01 (m, 1H), 6.98 (t, J = 7.4 Hz, 2H), 6.82 (s, 1H), 6.80 – 6.69 (m, 4H), 6.59 – 6.52 (m, 2H), 6.07 (s, 21H), 4.59 (s, 1H), 4.39 (s, 1H), 4.18 (s, 3H), 3.87 (q, J = 4.7 Hz, 1H), 3.02 – 2.95 (m, 1H), 2.80 (t, J = 11.6 Hz, 1H), 2.67 (d, J = 13.6 Hz, 2H), 2.22 – 2.02 (m, 3H), 1.91 (s, 1H), 1.81 (s, 3H), 1.84 – 1.74 (m, 2H), 1.24 (s, 1H).

B. (Cyclo)Ac-CFSRMC-NH_2

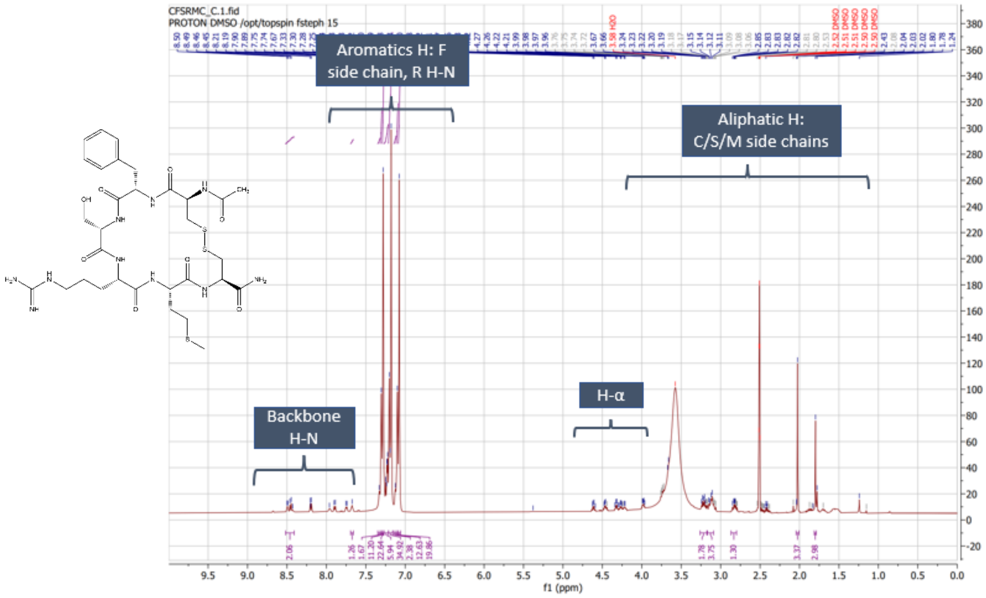

^{1}H NMR (500 MHz, DMSO) δ 8.52 – 8.41 (m, 2H), 7.67 (s, 1H), 7.33 (s, 2H), 7.30 (s, 11H), 7.28 (s, 23H), 7.27 – 7.21 (m, 6H), 7.19 (d, *J* = 12.0 Hz, 35H), 7.13 (s, 2H), 7.10 (s, 13H), 7.08 (s, 20H), 3.22 (dt, *J* = 11.9, 5.7 Hz, 2H), 3.17 – 3.09 (m, 4H), 2.87 – 2.80 (m, 1H), 2.02 (s, 3H), 1.80 (s, 3H).

C. (Cyclo)Ac-CLYHFC-NH_2

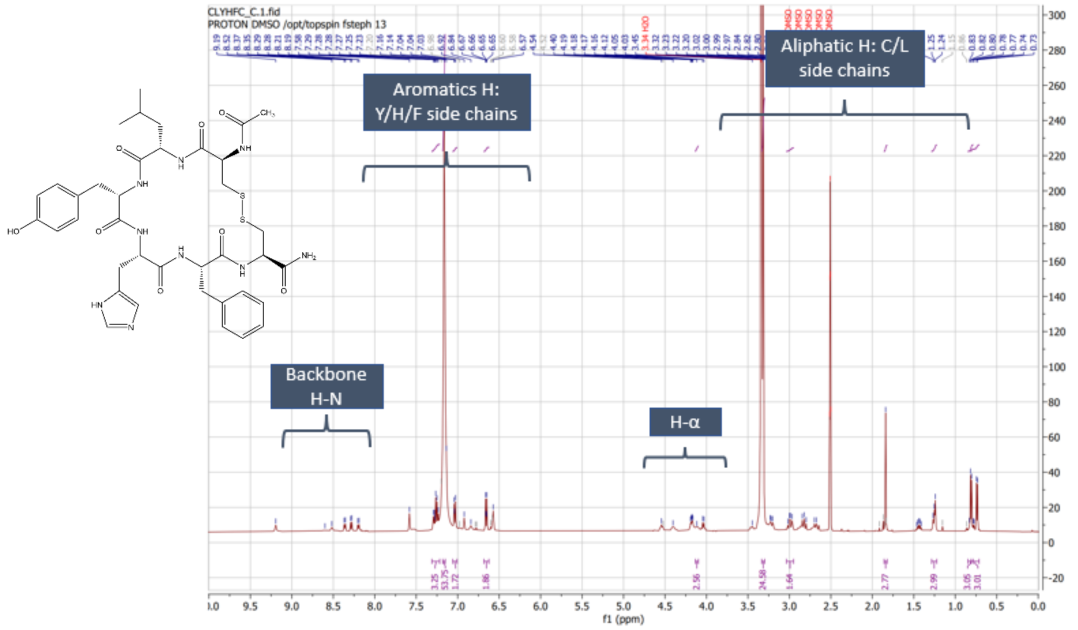

^{1}H NMR (500 MHz, DMSO) δ 7.26 (p, J = 7.2 Hz, 3H), 7.16 (s, 54H), 7.03 (d, J = 8.4 Hz, 2H), 6.68 – 6.62 (m, 2H), 4.12 (s, 3H), 3.32 (s, 25H), 2.99 (dd, J = 14.5, 10.7 Hz, 2H), 1.84 (s, 3H), 1.28 – 1.22 (m, 3H), 0.81 (d, J = 6.6 Hz, 3H), 0.81 – 0.71 (m, 3H).

D. (Cyclo)Ac-CYTYWC-NH_2

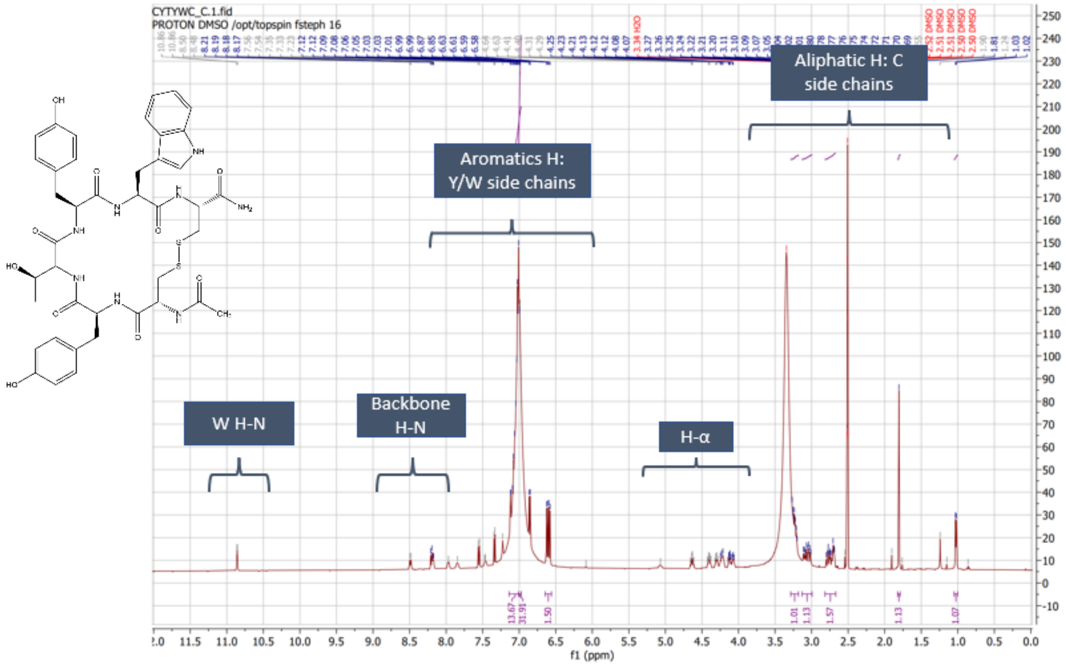

^{1}H NMR (500 MHz, DMSO) δ 7.14 – 6.97 (m, 14H), 6.99 (s, 32H), 6.60 (dd, J = 16.3, 8.4 Hz, 1H), 3.23 (tt, J = 12.7, 5.4 Hz, 1H), 3.06 (ddd, J = 29.6, 14.2, 6.2 Hz, 1H), 2.82 – 2.67 (m, 2H), 1.81 (s, 1H), 1.03 (d, J = 6.2 Hz, 1H).

E. (Cyclo)Ac-CLYKVC-NH_2

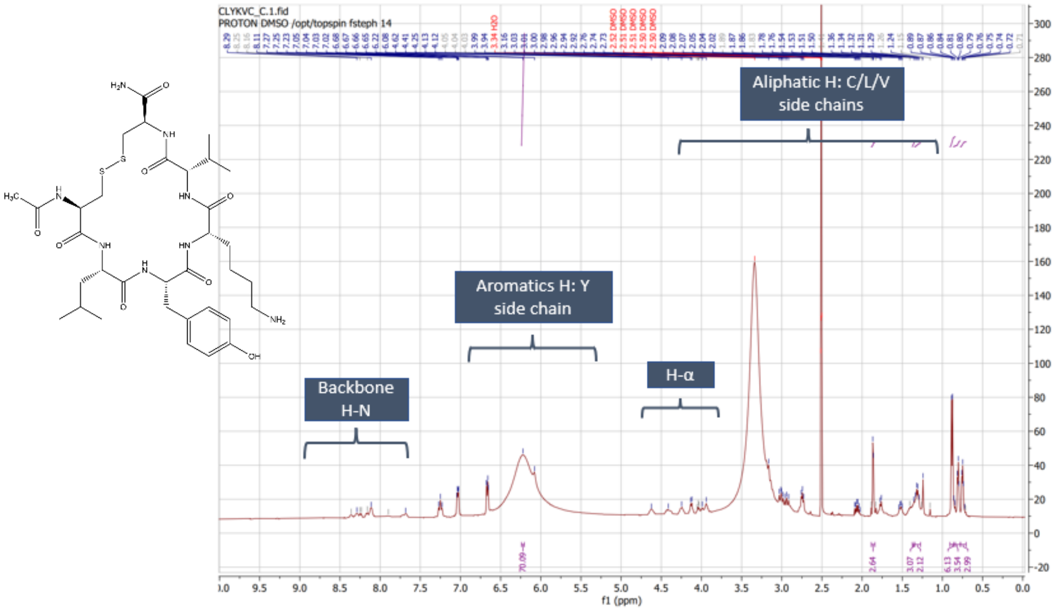

^{1}H NMR (500 MHz, DMSO) δ 6.22 (s, 70H), 1.86 (d, J = 4.3 Hz, 3H), 1.36 (s, 3H), 1.31 (q, J = 7.8 Hz, 2H), 0.88 (d, J = 6.7 Hz, 6H), 0.80 (t, J = 5.4 Hz, 4H), 0.75 (t, J = 5.0 Hz, 3H).

Table S3. Exact mass of the synthetic peptides

Peptide Sequence	Monoisotopic mass (Da)	$[M+H]^+$ found (LR-MS, Da)	Exact mass
Ac-CYYQWC-NH_2	905.32	906.13	905.13
(Cyclo-1,6)Ac-CYYQWC-NH_2	903.30	904.32	903.31
Ac-CFSRMC-NH_2	786.30	787.31	786.30
(Cyclo-1,6)Ac-CFSRMC-NH_2	784.28	785.29	784.28
Ac-CLYHFC-NH_2	825.33	826.17	825.16
(Cyclo-1,6)Ac-CLYHFC-NH_2	823.31	824.32	823.32
Ac-CYTYWC-NH_2	878.31	879.32	878.31
(Cyclo-1,6)Ac-CYTYWC-NH_2	876.29	877.31	876.30
Ac-CLYKVC-NH_2	768.37	769.40	786.40
(Cyclo-1,6)Ac-CLYKVC-NH_2	766.35	767.14	766.14
